# Yeast surface display of HIV Env protein variants for the discovery of novel vaccine immunogens

**DOI:** 10.1186/1742-4690-9-S2-P326

**Published:** 2012-09-13

**Authors:** S Grimm, M Ackerman

**Affiliations:** 1 Dartmouth College, Lebanon, NH, USA

## Background

The discovery of broadly neutralizing antibodies (bnAbs) such as 4E10, pg9 and VRC01 binding to the HIV env spike has electrified the field and shows that, in principle, the human immune system is capable of producing protective antibodies against HIV. All current vaccination strategies have however failed to elicit such bnAbs, leaving room for novel approaches to address this problem.

Protein engineering techniques such as yeast surface display have proven potential for the discovery and affinity maturation of monoclonal antibodies. Antibody libraries are presented on yeast cells and flow cytometric sorting is used to identify antibody variants with improved properties. As antibodies, also HIV env spikes are posttranslationally modified in the secretory pathway and composed of disulphide bond-containing glycoproteins. We here investigated whether HIV spike protein variants can be displayed or secreted from surface of the yeast strain S. cerevisiae.

## Methods

A panel of HIV env spike protein-encoding genes was inserted in vectors allowing for the display or secretion of the encoded proteins in S. cerevisiae. The displayed protein variants were analyzed for binding to either bnAbs or pooled HIV immunoglobulin from the serum of infected individuals (HIVIg) using flow cytometry. The secreted protein variants were tested for binding activities on an Octet biosensor.

## Results

We found that HIV env spike protein variants can be displayed or secreted from the yeast strain S. cerevisiae. Both displayed and secreted variants showed binding to bnAbs and HIVIg.

## Conclusion

This study suggests that yeast surface display is as a viable option for the engineering of HIV env spike protein variants and may become a valuable tool for the discovery of novel vaccine immunogens.

